# Novel Method for Border Irregularity Assessment in Dermoscopic Color Images

**DOI:** 10.1155/2015/496202

**Published:** 2015-10-29

**Authors:** Joanna Jaworek-Korjakowska

**Affiliations:** Department of Automatics and Biomedical Engineering, AGH University of Science and Technology, Aleja Mickiewicza 30, 30-059 Krakow, Poland

## Abstract

*Background*. One of the most important lesion features predicting malignancy is border irregularity. Accurate assessment of irregular borders is clinically important due to significantly different occurrence in benign and malignant skin lesions. *Method*. In this research, we present a new approach for the detection of border irregularities, as one of the major parameters in a widely used diagnostic algorithm the ABCD rule of dermoscopy. The proposed work is focused on designing an efficient automatic algorithm containing the following steps: image enhancement, lesion segmentation, borderline calculation, and irregularities detection. The challenge lies in determining the exact borderline. For solving this problem we have implemented a new method based on lesion rotation and borderline division. *Results*. The algorithm has been tested on 350 dermoscopic images and achieved accuracy of 92% indicating that the proposed computational approach captured most of the irregularities and provides reliable information for effective skin mole examination. Compared to the state of the art, we obtained improved classification results. *Conclusions*. The current study suggests that computer-aided system is a practical tool for dermoscopic image assessment and could be recommended for both research and clinical applications. The proposed algorithm can be applied in different fields of medical image analysis including, for example, CT and MRI images.

## 1. Introduction

**Figure 1:**
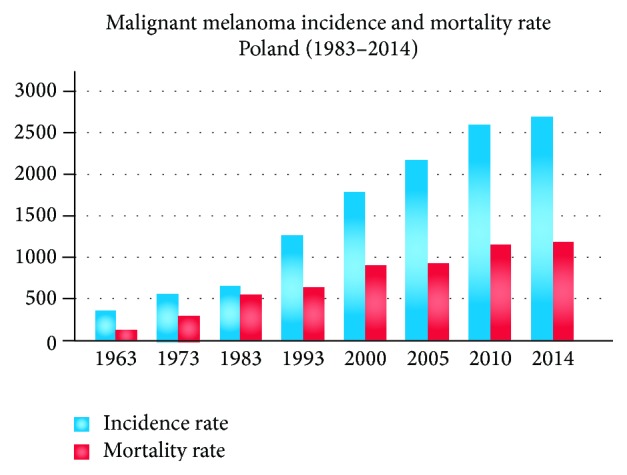
Statistics of malignant melanoma incidence and mortality rate over the past 50 years. Clinical data based on [25].

The skin is the body's largest organ that covers the entire body and protects it against infection, sunlight, and injury. Similar to other parts of the body, the cancer may occur also in the skin. Skin cancer is the most commonly diagnosed type of cancer in all people, regardless of age, gender, or race [1]. The most malignant type of skin cancer is melanoma. Malignant melanoma (latin:* melanoma malignum*) originates in pigment producing cells called melanocytes, which derive from neural crest [1–3]. Melanomas are fast-growing and highly malignant tumors often spreading to nearby lymph nodes, lungs, and brain. Malignant melanoma is likely to become one of the most common malignant tumors in the future, with even a ten times higher incidence rate [3].  Figure 1 presents the growth of incidence and mortality rate for malignant melanoma in Poland during the last 50 years.

Due to the high skin cancer incidence, dermatological oncology has become a quickly developing branch of medicine. Nowadays the progress is visible both in primary research concerning pathogenesis of tumors (the role of genes or viruses in tumor development) and in the development of new, more efficient methods of computer-aided diagnosis [4].

This paper is organized in 4 sections as follows.  Section 1 (Introduction) presents the problem of skin cancer and describes the clinical definition of border irregularity, motivation, and related works.  Section 2 (Materials and Methods) specifies the border irregularity assessment algorithm, including preprocessing, segmentation, borderline calculation, and irregularities detection. In Section 3 (Results and Discussion) the conducted tests and results are described.  Section 4 (Conclusions) closes the paper and highlights future directions.

**Figure 2:**
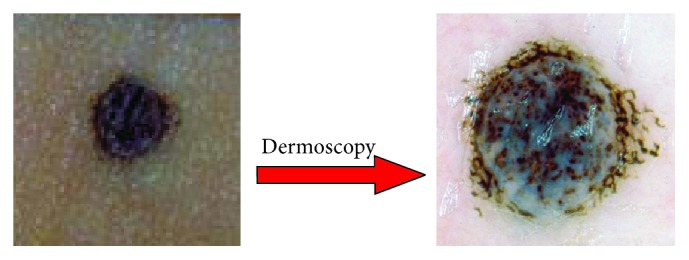
Dermoscopy enables clinicians to observe border irregularities, colors, and structures within skin lesions that are otherwise not visible to the unaided eye.

*Clinical Definition and Motivation*. The examination of the small moles is possible through a digital epiluminescence microscopy ((ELM), also dermoscopy or dermatoscopy) which is a noninvasive, in vivo technique which, by employing the optical phenomenon of oil immersion, makes subsurface structures of the skin accessible for examination and thus provides the additional criteria for the clinical diagnosis of pigmented skin lesion [1, 5]. ELM uses an optic magnification to visualize the features that are invisible to the naked eye (as shown in Figure 2).

Border irregularity is one of the leading parameters in the widely used diagnostic algorithm ABCD rule of dermoscopy which is one of the first proposed diagnostic algorithms and it is one of the most commonly used methods for evaluation of melanocytic lesions. The ABCD rule was originally proposed in 1994 by Stolz and coworker [1]. Nachbar et al. proved the reliability of the ABCD rule in a prospective study [6]. For 172 melanocytic lesions (69 melanomas and 103 melanocytic nevi) specificity was 90.3% and sensitivity was 92.8% [1, 6]. In order to calculate the ABCD score, the asymmetry, border irregularity, amount of colors, and differential structures have to be assessed semiquantitatively.

A skin lesion having ragged, notched, or blurred edges is very suspicious and mostly gives the first warning sign for melanoma. The irregularity is detected when a sharp, abrupt cut-off of pigment pattern appears at the periphery of the skin mole. After assessing ABCD parameters, each of the criteria has to be multiplied by a given weight factor yielding a total dermoscopy score (TDS) according to Table 1. The result of total dermoscopy score (TDS) predicts if the lesion is benign or malignant. TDS value less than 4.75 indicates a benign melanocytic lesion, the value between 4.8 and 5.45 indicates a suspicious lesion, and the value greater than 5.45 is highly suspicious for melanoma [4].

*Related Works*. Biomedical researchers observe that clinicians have difficulties in the correct visual assessment of border irregularity and that their calculations are not invariant to reflection and rotation. Due to this obstacles researchers try to improve the calculation of the border irregularity and make the assessment of the skin mole much easier. Nowadays, a widely used method to reflect the borderline is the estimation of the radial distance between the center of the mass and the borderline [7]. The centroid distance curve establishes the projection from the angular orientations to the sum of the lengths of those line segments connecting the lesion centroid and border points [7]. This approach is of great interest but still may lead to many errors especially when segmented area is not round. Comparison between the proposed method and radial distance has been done in Section 3 (Results and Discussion).

Besides the radial distance method different parameters have been proposed to estimate the irregularity such as compactness index, solidity, fractal dimensions, and indentation irregularity index. In [8], authors presented the state of the art in computerized analysis of pigmented skin lesions describing also the border detection methodology.  Table 2 contains references to articles aimed at computing descriptors for border irregularity evaluation.

## 2. Materials and Methods

**Figure 3:**
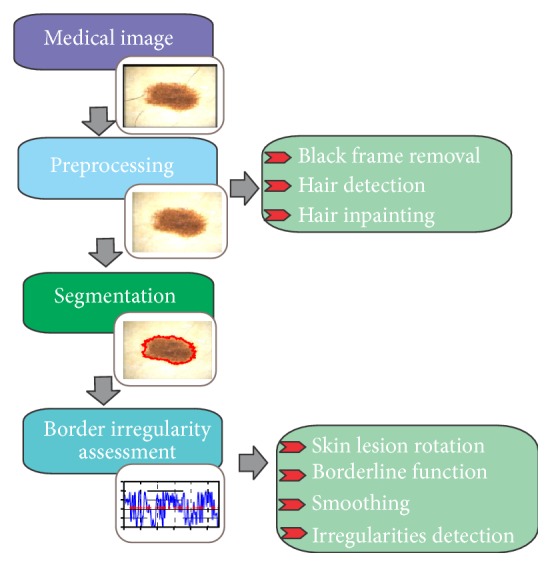
Proposed method for the border irregularity assessment in dermoscopic color images.

In this paper, we propose a new automatic system for the assessment of border irregularities. The overview of the steps is presented in Figure 3.

The system is divided into four main stages, including preprocessing (image enhancement), segmentation, and border irregularities assessment. The last stage contains few smaller steps containing skin lesion rotation, borderline function, smoothing, and irregularities detection.

In this section, the preprocessing and segmentation steps are described shortly, based on previous works, while the main stage which is border irregularity assessment is presented in detail.

### 2.1. Dermoscopic Image Preprocessing

The first step in every medical image processing system is the image acquisition, which aims at obtaining an image of the best quality. After a medical or dermoscopic image is acquired, it may not have the expected quality to perform the diagnostic analysis. Therefore, in most cases, the next stage is called preprocessing and it is responsible for reducing the amount of artifacts and noise in the image. For dermoscopy images the preprocessing step is obligatory, because of extraneous artifacts, such as skin lines, air bubbles, and hairs which appear in almost every image. The preprocessing stage consists of two parts. The first step is the removal of black frame that is introduced during the digitization process. The second step is a hair-removal algorithm which comprises two steps: hair detection and inpainting.

#### 2.1.1. Black Frame Removal

In the first step, we remove the black frame which is introduced during the digitization process (normal output from some types of dermatoscope). In order to determine the darkness of a pixel with (*R*; *G*; *B*) coordinates, the lightness component of the HSL color space is calculated as follows [9–11]:

(1) $$\begin{array}{c} L=\frac{\max\left(R,G,B\right)+\min\left(R,G,B\right)}{2}. \end{array}$$

A pixel is considered to be black when the lightness value is less than 15 (range of the lightness value [0 : 255]). We scan rows in four directions (top, bottom, right, and left) and calculate the amount of black pixels. A particular row is labeled as the part of the black frame if it contains 50% of black pixels and is being removed. Additionally, we remove 10 more rows that represent the light-colored part of the frame.

#### 2.1.2. Hair Detection and Inpainting

The removal and restoration of dark, thick hairs and hair-like regions within the skin lesion images have to be done separately and it is required for effective segmentation and classification of border irregularities. A number of methods have been developed for hair removal in dermoscopic images and they were mostly based on morphological operations and adaptive thresholding [12–15]. A good approach for hair removal is the use of top-hat transform. The process consists of four steps. Firstly, we convert the dermoscopic *RGB* image into grayscale with the NTSC 1953 standard. Secondly, black top-hat transform is used for the detection of dark, thick hairs:

(2) $$\begin{array}{c} T_{w}\left(\;I\;\right)=I\circ b-I, \end{array}$$

where ∘ denotes the closing operation, *I* is the grayscale input image, and *b* a grayscale structuring element [16].

**Figure 4:**
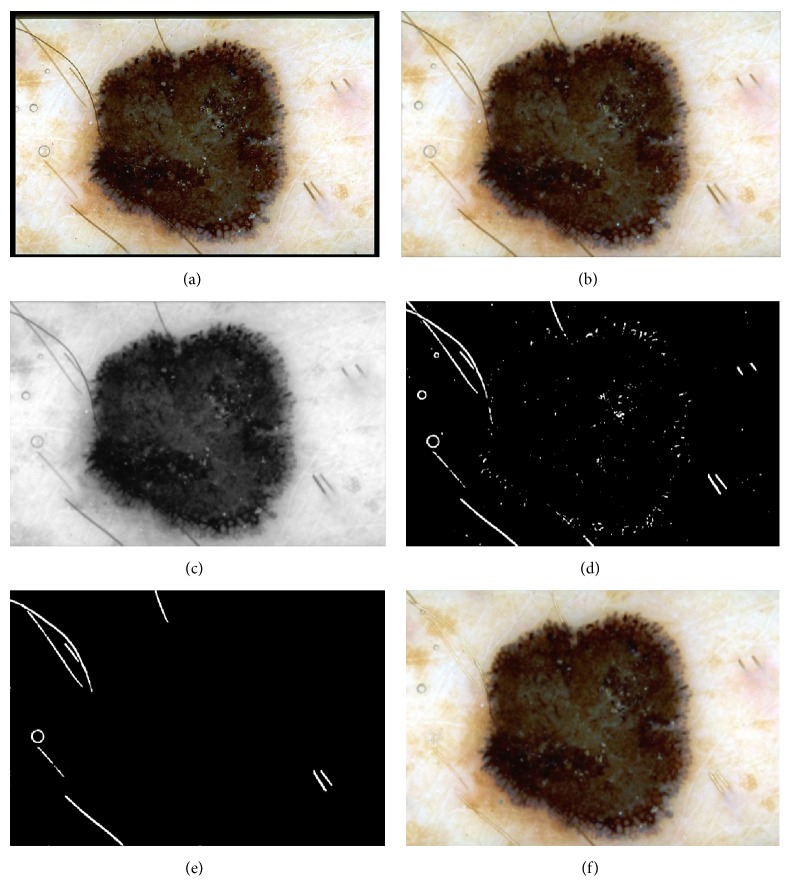
Outcome of the preprocessing step: (a) input image, (b) black frame removal, (c) grayscale conversion, (d) top-hat transform and binarization process, (e) hair distinction from other structures, and (f) inpainting.

The black top-hat transform is defined dually as the difference between the closing and the input image. The aim of the next step is to distinguish hairs from other local structures, such as dots and globules and pigment network that could also be detected by the top-hat transform. This is achieved by comparing the areas, major and minor axes, and perimeters [10]. Hair line pixels are replaced with the values calculated on the basis of the neighborhood pixels (average value) [10, 17].  Figure 4 presents the outcome after each step, including the black frame removal and hair inpainting.

### 2.2. Skin Lesion Segmentation

**Figure 5:**
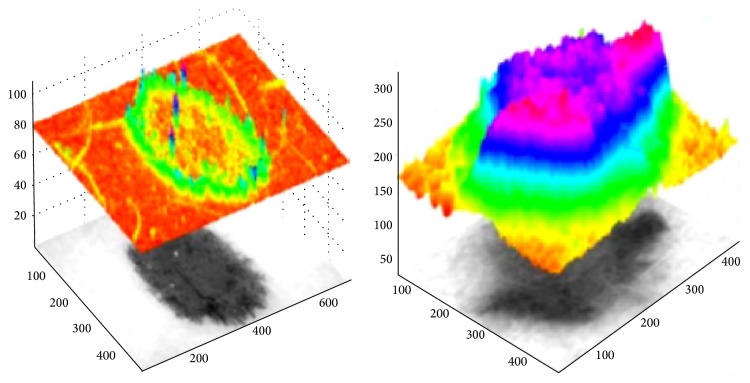
Intensity analysis of dermoscopy images converted to grayscale.

The segmentation process in one of the most important and challenging steps in dermoscopic image processing. It has to be fast and accurate, because the subsequent steps crucially depend on it. The segmentation process for dermoscopic images is extremely difficult due to several important factors: low contrast between the healthy skin and the mole, variegate coloring inside of the lesion, irregular borders, and different artifacts [8]. Automatic extraction of lesion is not a trivial task, because the skin lesion has mostly nonuniform coloring, and the surrounding is covered with the remaining parts after the preprocessing step which make the process even harder to carry out. Therefore, the segmentation algorithms are one of the most active areas in the dermoscopy image analysis. Due to the difficulties described above numerous methods have been implemented and tested. Celebi et al. present in their research [18] the state of the art of segmentation methods and compare them with the statistical region merging as a recent color image segmentation technique based on region growing and merging. In our research we have implemented and tested many different segmentation algorithms. On the grounds of the results and experiments the skin lesion extraction is based on seeded region-growing algorithm [17], in regard to two aspects. During the preprocessing step, after smoothing and hair removal, the healthy skin becomes homogeneous. Secondly, analyzing the border irregularity, the whole skin lesion has to lie inside the dermoscopic image. It means that the healthy skin surrounds the mole. Region growing techniques generally give better results in noisy images where edges are extremely difficult to detect. In Figure 5, we present the verification of our assumption that region growing algorithm accomplished on the healthy skin area will achieve satisfactory results.

The aim of the image segmentation stage is to extract the lesion area from the healthy skin. In general, segmentation process divides the image *I* into two regions *S*_*i*_:

(3) ⋃i=12Si=I,S1∩S2=∅.

**Figure 6:**
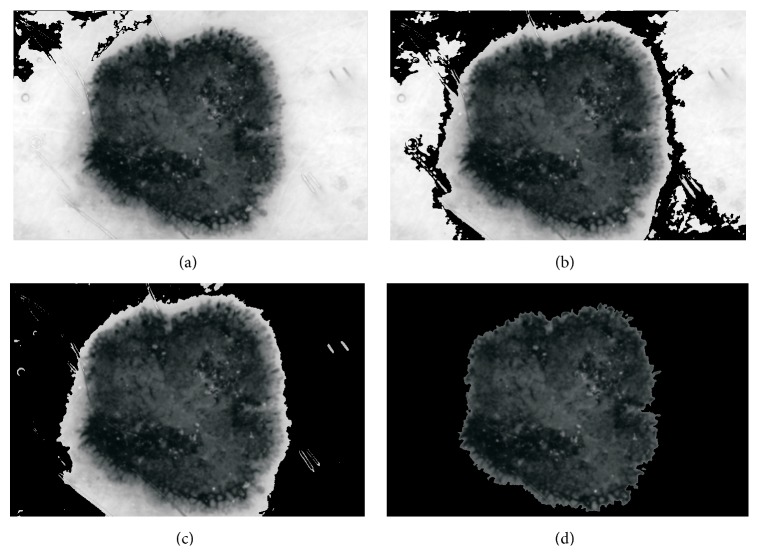
Results of the segmentation process: (a–c) segmentation of the healthy skin with the region-growing algorithm and (d) segmented area (healthy skin: black color, skin mole: remaining grayscale area).

For the skin lesion segmentation we take one seed which is located in the left upper corner of the image. The region is iteratively grown by comparing all unallocated neighboring pixels to the region. The region growing process consists of picking a seed from the set, investigating all 4-connected neighbors of this seed, and merging suitable neighbors to the seed. The seed is then removed and all merged neighbors are added to the seed set. The region growing process continues until the seed set is empty. We define *δ*(*x*) to be a measure of how different *x* is from the region it adjoins. The difference between a pixel's intensity value and the region's mean, *δ*, is used as a measure of similarity. The pixel with the smallest difference measured in this way is allocated to the respective region. This process continues until all pixels are allocated to a region. In Figure 6 the results of subsequent steps of the segmentation process are presented.

### 2.3. Skin Lesion Rotation

**Figure 7:**
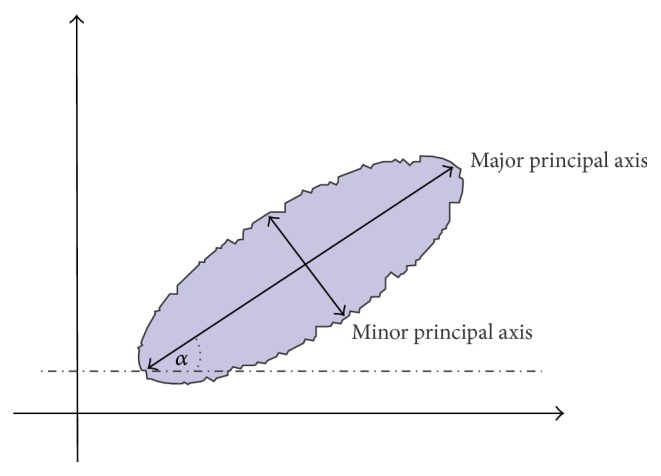
Proposed method for the border irregularity assessment: rotation axis and rotation angle.

Skin lesion rotation is the first step of the border irregularity assessment algorithm. In order to calculate the most reliable borderline function we have to rotate the image so that the major axis of the skin lesion is parallel to the horizontal line as shown in Figure 7. Rotation takes place entirely in the spatial domain. Spatial transforms involve remapping of one set of pixels (i.e., image) to another. In this regard, the original image can be considered the input to the remapping process and the transformed image is the output of the rotation process [19]. If images were continuous, then remapping would not require interpolation, but the discrete nature of pixels usually necessitates remapping (except rotation by 90 or 180 degrees) [19].

Transformation of an image *I* defined over a (*w*, *z*) coordinate system to an image *I*_*g*_ defined over an (*x*, *y*) coordinate system can be defined as

(4) $$\begin{array}{c} I_{g}\left(\;x,y\;\right)=T\left\{\;I\left(\;w,z\;\right)\;\right\}, \end{array}$$

where *T* is the rotation transformation [19].

In two dimensions, to carry out a rotation using matrices the point (*x*, *y*) to be rotated (orientation from positive *x* to *y*) is written as a vector and then multiplied by a matrix calculated from the angle, *θ*:

(5) $$\begin{array}{c} \left[\begin{array}{c} x\\ y \end{array}\right]=\left[\begin{array}{cc} \cos\theta & -\sin\theta \\ \sin\theta & \cos\theta \end{array}\right]\left[\begin{array}{c} w\\ z \end{array}\right], \end{array}$$

where (*x*, *y*) are the coordinates of the point after rotation [19].

For the interpolation, the bilinear method instead of nearest neighbor method has been chosen. The nearest neighbor interpolation method is faster than bilinear interpolation but, however, produces results inferior to those obtained with bilinear interpolation [20]. In the bilinear interpolation method, the output pixel is the weighted average of transformed pixels in the nearest 2 by 2 neighborhood.

**Figure 8:**
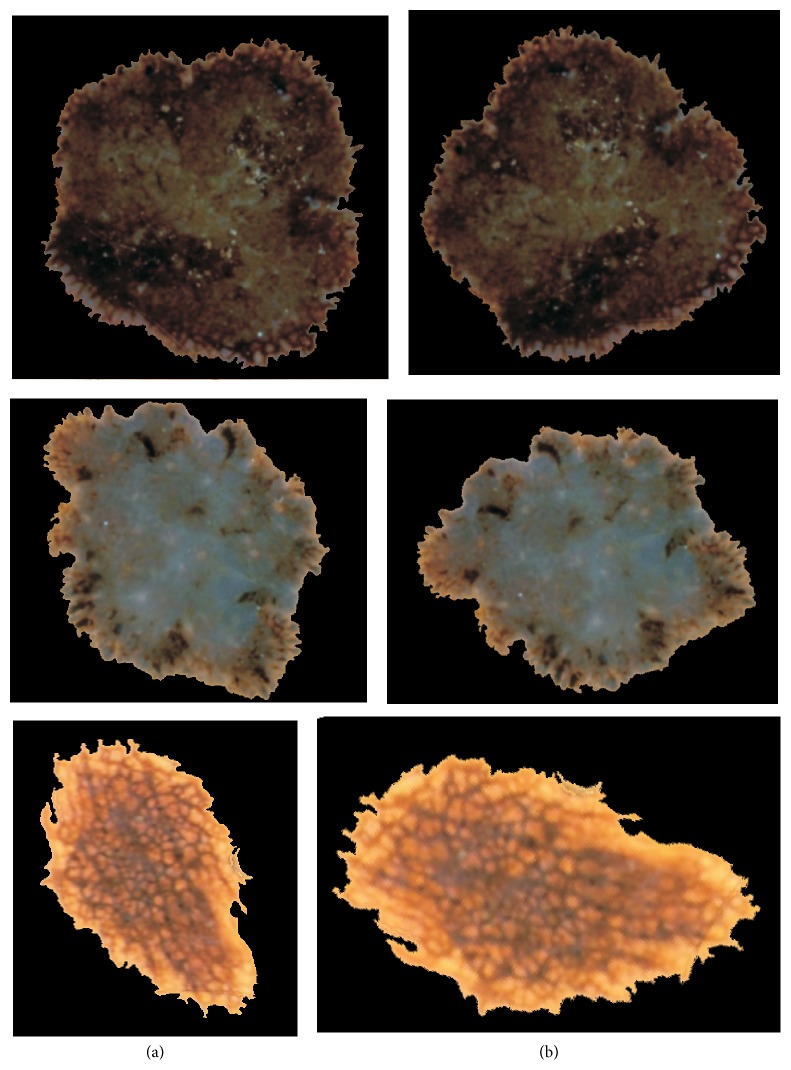
Proposed method for the rotation of the skin lesion. On the left side: (a) the outcome of the segmentation step; on the right side: (b) the result of the skin lesion rotation.

The skin lesion rotation has a large impact on the borderline function calculation. It gives the opportunity to analyze the borderline precisely detecting each irregularity with high accuracy.  Figure 8 presents results of the rotation function for three examples.

### 2.4. Borderline Function

**Figure 9:**
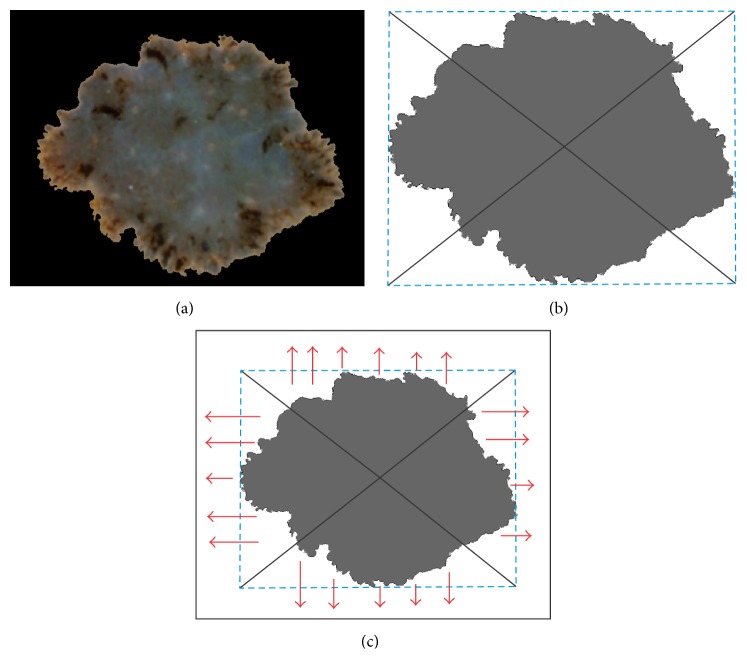
Proposed method for the borderline function calculation: (a) skin lesion after rotation, (b) bounding box and lines connecting center of the mass with vertices, and (c) directions in which the distances between the border and the image edge are calculated.

After rotating the skin lesion the borderline function can be calculated. Firstly, we trace the exterior boundaries of the segmented object. Secondly, we calculate the bounding box of the object and the center of the mass as shown in Figure 9(b). Bounding box is in geometry described as a minimum bounding rectangle enclosing a point set (*S*) in *N* dimensions which is the box with the smallest measure. Finally, we find the boundary pixels lying on the lines connecting the center of the mass with the vertices. These four points make it possible to divide the boundary into four parts which are not the same length. The most important fact is that the irregularities are arranged in the right direction as illustrated in Figure 9(c). In the next step we calculate the distance between the border and the image edge (Figure 9(c), red arrows) for each part of the borderline [21].

**Figure 10:**
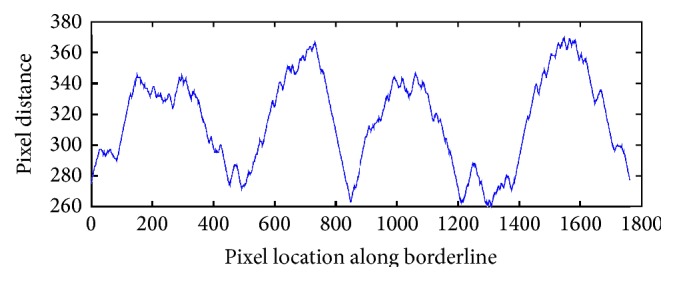
Borderline function received after applying the described algorithm.

Before we receive the final version of the borderline function, we have to subtract the gap between the functions which result from the difference in distance to the edge. As a result of the calculation we obtain a function with an exact reflection of the border irregularities (Figure 10).

### 2.5. Smoothing

For the determination of the ragged edges we smooth the signal with Gaussian filter. The Gaussian smoothing operator is a convolution operator that is used to “blur” signals or images and remove detail and noise. In this sense, it is similar to the mean filter, but it uses a different kernel that represents the shape of a Gaussian (“bell-shaped”) hump. This kernel has some special properties which are detailed below:

(6) $$\begin{array}{c} f\left(\;x\;\right)={\frac{1}{\sqrt{2\pi \sigma }}e}^{-x^{2}/2\sigma^{2}}, \end{array}$$

where *σ* is the standard deviation of the distribution.

**Figure 11:**
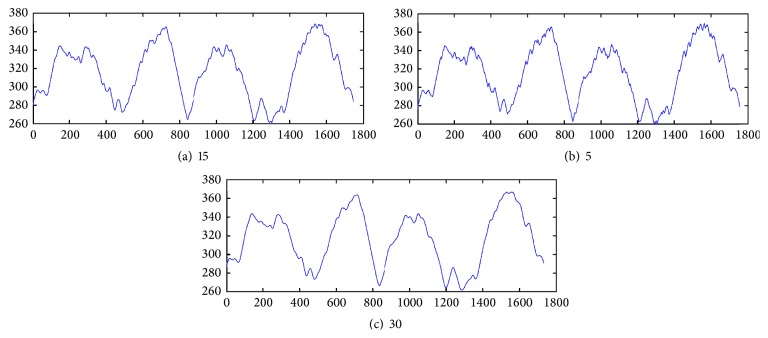
Outcome of the Gaussian low-pass filter of different size.

Gaussian smoothing filter gives more weight at the central pixels and less weights to the neighbors.  Figure 11 shows differences for various filter size and *σ* = 0.5. Through experimental studies we have selected the Gaussian low-pass filter of size 15.

### 2.6. Border Irregularities Detection

In mathematics, a stationary point (also called critical point) of a differentiable function of one variable is a point of the domain of the function where the derivative is zero. A special group of stationary points are turning points. A turning point is a point at which the derivative changes sign.

A turning point may be either a relative maximum or a relative minimum. If the function is differentiable, then a turning point is a stationary point. Border irregularities appear as turning points in the borderline function. Therefore, we calculate the derivative of the borderline function to find local maximum points of the function. The local maximum is detected when the derivative of the function crosses the zero point and the slope changes sign from + to −.

The necessary conditions are as follows:

(7) f′x0=0,f′x>0for  x∈x0−δ,x0,f′x<0for  x∈x0,x0+δ.

**Figure 12:**
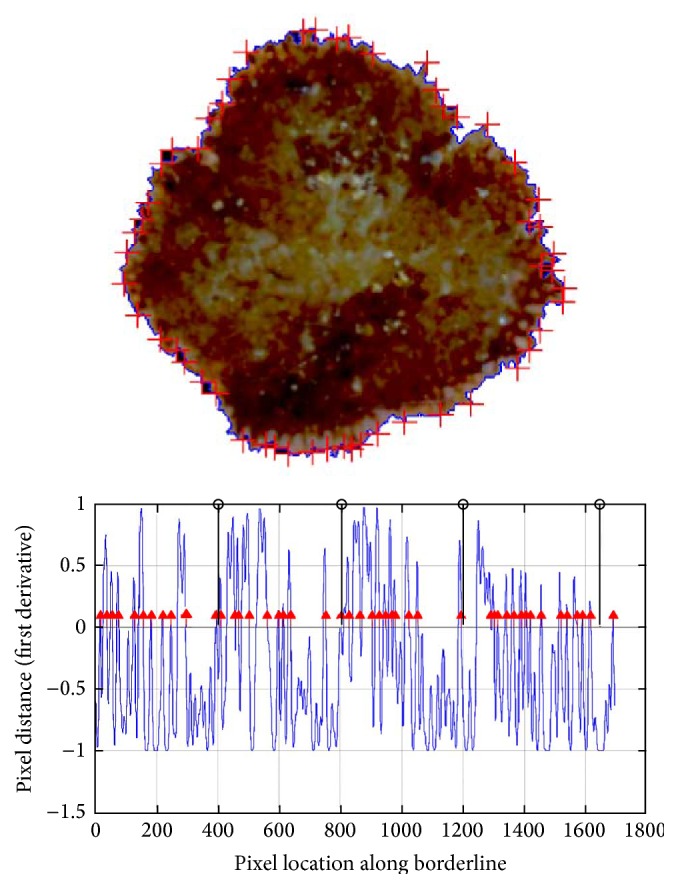
Result of the described border irregularity assessment algorithm for a dermoscopic image. With the red arrows the detected irregularities are marked. The black lines divide the borderline function into four equal parts for further classification.

Figure 12 presents the outcome of the border irregularities detection algorithm.

## 3. Results and Discussion

### 3.1. Database Specification

The proposed and implemented algorithm for the diagnosis of the pigmented skin lesion has been tested on dermoscopic images provided by two university hospitals (University of Naples, Italy, and University of Graz, Austria), where they were stored on a CD-ROM in JPEG format [1] and by a private database. The documentation of the dermoscopic images was performed using a Dermaphor apparatus (Heine, Optotechnik, Herrsching, Germany) and a photo camera (Nikon F3) mounted on a stereomicroscope (Wild M650, Heerbrugg AG, Switzerland) in order to produce digitized ELM images of skin lesions. All of the images were assessed manually by one dermoscopic expert with an extensive clinical experience (10 years).

Furthermore, all of the cases were based on histopathological examination of the biopsy material. For this study, because of the assumptions resulting from the border irregularity diagnostic algorithm, we have chosen images that fitted entirely in the image and did not contain too many hairs in the examined lesion. The dermatology expert has chosen images which were possible to be evaluated in terms of border irregularity. In order to develop and test the automatic procedure for the diagnosis of pigmented skin lesions, 350 images containing 280 benign and 70 malignant cases with different resolutions, ranging from 0.033 to 0.5 mm/pixel, were chosen.

**Figure 13:**
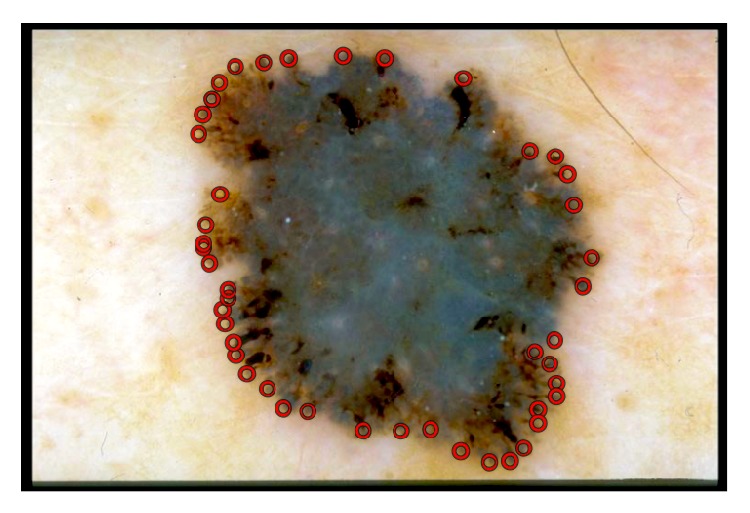
Example of manual selection of border irregularities performed by an experts.

The experts assessed every image in terms of the border irregularity (Figure 13). The database contains 140 cases with border irregularity less than 3 and 210 skin lesions with border irregularity above 4. The preprocessing step (black frame removal and hair removal) as well as the segmentation step (border error less than 6%) did not affect the further research.

### 3.2. Statistical Analysis

For the evaluation of the correctness of the implemented border irregularity detection algorithm, we use sensitivity and precision which are defined in terms of systematic and random errors. In this study case, it is hard to define the true negative value; thus, the accuracy is impossible to calculate.

Sensitivity, also known as True Positive Rate (TPR) or recall, is the proportion of border irregularities that tested positive and are positive (True Positive (TP)) of all the irregularities that actually are positive (Condition Positive, CP = TP + FN). It can be seen as the probability that the test is positive given that the border part is irregular. With higher sensitivity, fewer actual cases of irregularities go undetected. Sensitivity is defined by (8)

(8) $$\begin{array}{c} \text{Sensitivity}=\frac{\text{TP}}{\text{TP}+\text{FN}}. \end{array}$$

On the other hand, we calculate precision (positive predictive value) that is defined as the proportion of the true positives against all the positive results (both true positives and false positives). Precision is defined by

(9) $$\begin{array}{c} \text{Precision}=\frac{\text{TP}}{\text{TP}+\text{FP}}. \end{array}$$

The border irregularity detection is in many cases hard to assess. We only check the border irregularities that were obvious for the physician and compare them manually with our results. The proposed algorithm achieved average sensitivity of 91% and precision of 89% (Table 3).

### 3.3. Comparison with Other Related Studies

**Figure 14:**
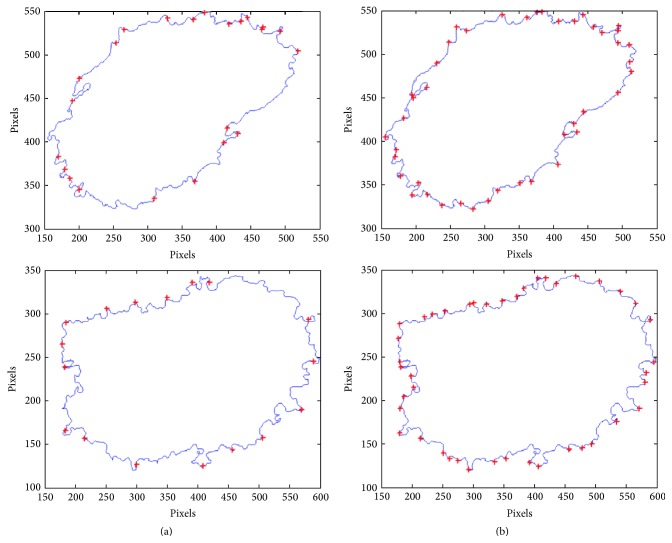
Comparison of border irregularities measured for the same skin lesion (a) with the radial distance and (b) with the proposed algorithm.

Table 2 lists the state of the art of previous studies proposed for the successful assessment of border irregularities. However, only the radial distance method enables the reflection of the borderline. Other described parameters present only a single value which depicts the roundness and smoothness of the skin mole. For the evaluation of the advantages and correctness of the implemented algorithm, we present few outcomes after applying the radial distance algorithm and the described solution (Figure 14).

## 4. Conclusions

One of the main tasks of modern dermatology is the detection of melanoma in its early stage of development, because the survival rate after identification of less than 0.75 mm thick melanomas is nearly 100% [1, 2]. This can be achieved by combining new instruments with the computer-aided diagnosis [22, 23]. The aim of these systems is to increase the specificity and the sensitivity of melanoma recognition and reduce unnecessary biopsies [24]. Amount of border irregularities is one of the most important factors in the assessment of malignancy of skin moles. In this paper, a new method for the border irregularities detection has been proposed. The implemented algorithm obtained better results for border irregularities appearing not radially compared to the approaches described in the literature. The proposed algorithm can be part of a sophisticated screening system. Screening systems can be used not only by young inexperienced dermatologist but first and foremost by family physicians, which can contribute to the early detection of the melanoma. Regular checkups play a vital role in allowing for the early detection of melanoma. Screening systems also provide a new opportunity for people living in remote and rural areas outside the regional center and thus having difficulties in making an appointment with a dermatologist.

## Figures and Tables

**Table 1:** ABCD rule criteria (based on [4]).

Parameter	Definition	Score	Weight
A-asymmetry	Number of asymmetry axess	0–2	1.3

B-border	Number of octanes with irregularity	0–8	0.1

C-color	Number of colors	1–6	0.5

D-differential structures	Presence of network, structureless, or homogeneous areas, streaks, dots, and globules	1–5	0.5

**Table 2:** Overview of the references for border irregularity descriptors (based on [8]).

Border irregularity descriptors	Computer vision references
Compactness index	[26–28]
Convex hull (solidity)	[29, 30]
Fractal geometry and dimensions	[31–33]
Irregularity index	[34, 35]
Fourier features	[36]
Wavelet transform	[37]

**Table 3:** List of parameters evaluated by the proposed method.

Number of cases	Number of irregularities	Sensitivity [%]	Precision [%]
140	0–3	86	81
57	4–10	89	91
116	10–20	93	88
37	>20	96	94
Average		91	89
